# Characterization of a Large Panel of Rabbit Monoclonal Antibodies against HIV-1 gp120 and Isolation of Novel Neutralizing Antibodies against the V3 Loop

**DOI:** 10.1371/journal.pone.0128823

**Published:** 2015-06-03

**Authors:** Yali Qin, Saikat Banerjee, Aditi Agrawal, Heliang Shi, Marisa Banasik, Feng Lin, Kari Rohl, Celia LaBranche, David C. Montefiori, Michael W. Cho

**Affiliations:** 1 Department of Biomedical Sciences, Iowa State University, Ames, IA, 50011, United States of America; 2 Center for Advanced Host Defenses, Immunobiotics and Translational Comparative Medicine, Iowa State University, Ames, IA, 50011, United States of America; 3 Department of Surgery, Duke University, Durham, NC, 27710, United States of America; Shanghai Medical College, Fudan University, CHINA

## Abstract

We recently reported the induction of potent, cross-clade neutralizing antibodies (nAbs) against Human Immunodeficiency Virus type-1 (HIV-1) in rabbits using gp120 based on an M-group consensus sequence. To better characterize these antibodies, 93 hybridomas were generated, which represent the largest panel of monoclonal antibodies (mAbs) ever generated from a vaccinated rabbit. The single most frequently recognized epitope of the isolated mAbs was at the very C-terminal end of the protein (APTKAKRRVVEREKR), followed by the V3 loop. A total of seven anti-V3 loop mAbs were isolated, two of which (10A3 and 10A37) exhibited neutralizing activity. In contrast to 10A3 and most other anti-V3 loop nAbs, 10A37 was atypical with its epitope positioned more towards the C-terminal half of the loop. To our knowledge, 10A37 is the most potent and broadly neutralizing anti-V3 loop mAb induced by vaccination. Interestingly, all seven anti-V3 loop mAbs competed with PGT121, suggesting a possibility that early induction of potent anti-V3 loop antibodies could prevent induction of more broadly neutralizing PGT121-like antibodies that target the conserved base of the V3 loop stem.

## Introduction

A critical problem for developing a vaccine against human immunodeficiency virus type 1 (HIV-1) is the difficulty in inducing broadly neutralizing antibodies (bnAb) against the large number of viral variants that exist [1–3]. The envelope glycoproteins gp120 and gp41 are the sole HIV-1 antigens on the virion surface targeted by nAbs. Therefore, characterizing the immunogenic and structural features of the HIV-1 envelope is important for designing immunogens to elicit bnAbs and to understand the humoral response to HIV-1 infection [4–6].

Monoclonal antibodies (mAbs) have been important tools for probing antigen structures. Recent technology developments for antigen-specific single B cell sorting [7,8], high-throughput clonal memory B-cell cultures [9] and next-generation sequencing (NGS) [10] have enabled isolation of a large number of new bnAbs against HIV-1 from virus-infected patients [11]. Those bnAbs have defined four major targets on the HIV-1 envelope: the CD4 binding site (CD4BS), glycans around N160 along with conserved elements on V1/V2, the base of and glycans around the V3 loop, and the membrane-proximal external region (MPER) of gp41 (as reviewed in [12,13]). Recently, epitopes involving both gp120 and gp41 have been identified as well [14–17].

In contrast to bnAbs isolated from HIV-1 infected humans, envelope-specific mAbs generated from vaccinated subjects, either animals or humans, are limited. Early studies isolated many murine mAbs from immunized animals. However, most did not possess significant neutralizing activity [18–23]. Later, Gao *et al*. reported two mAbs isolated from gp140-immunized mice that cross-reacted with all tested envelope proteins, but neither mAb neutralized primary HIV-1 pseudoviruses [24]. Derby *et al*. isolated six anti-gp120 mAbs from mice immunized with soluble gp140 [25]. These antibodies could neutralize the homologous SF162, and their activities were dependent on the glycosylation patterns of the V1, V2 or V3 loops. However only one anti-V3 mAb displayed cross-clade neutralizing activity, which was dependent on the type of V1 loop present on heterologous viruses. Recently, Sundling *et al*. used a non-human primate (NHP) model to evaluate envelope immunogens that elicited anti-CD4BS antibodies, isolating a panel of functional mAbs from immunized rhesus macaques [26]. However, only eight mAbs were generated that exhibited neutralizing activity against a limited number of mostly tier 1 HIV-1 isolates (*viz*. MN.3, HXBc2, SF162 and MW965.26). The RV144 clinical trial reported an estimated 31% protection efficacy, and protection correlated with the presence of anti-V2 loop antibodies in immunized individuals [27–29]. Most recently, four V2 mAbs were isolated from RV144 participants [30]. These mAbs recognized amino acid residue 169, neutralized tier 1 laboratory HIV-1 strains and mediated antibody dependent cell-mediated cytotoxicity (ADCC). Additionally, two other neutralizing V3 loop mAbs (CH22 and CH23) were isolated from the participants in RV135 trial, which showed limited neutralization against tier 1 strains [31].

Rabbits have been used widely in HIV-1 vaccine studies. Compared to other animal models, rabbits are large enough for collection of sufficient amount of antisera for nAb assessment, and can develop long CDR3s [4,24]. Although rabbits have restricted antibody germline usage, particularly in the heavy chain [32,33], their immune system is similar enough to that of humans [34–38] to allow for assessment of potential nAb induction. Furthermore, rabbit hybridomas can be generated to produce mAbs to determine the specific characteristics of individual antibodies. Recently, Chen *et al*. [39] reported generation and characterization of twelve mAbs from HIV-1 Env-immunized rabbits, three of which exhibited neutralizing activity.

We recently reported induction of potent neutralizing antibodies using soluble gp120 based on M group consensus sequence (MCON6; [40,41]) in rabbits [42]. Although the primary neutralizing epitope appeared to be the V3 loop and that neutralizing activity was largely against tier 1 virus isolates, the cross-clade neutralizing breadth was substantial. Further characterization of antibodies against gp120 in a follow up study revealed that some of them could compete with bnAbs VRC01, PGT121, and PGT126 in binding gp120, suggesting that the antibodies bound at or near the vicinity of epitopes targeted by these bnAbs [43].

Although we did not succeed in inducing “true” bnAbs using our vaccine regimen, it was important to (1) further characterize the nature of potent neutralizing activity against the V3 loop, (2) identify target epitopes of antibodies that competed with bnAbs VRC01 and PGT121/126, (3) determine immunogenic epitopes of antibodies that failed to exhibit neutralizing activity in order to develop better immunogens or immunization strategies, and (4) examine maturation pathways of these antibodies. To achieve these objectives, antibodies will have to be characterized at a clonal level. In this study, we generated 93 hybridomas from one of the gp120-immunized rabbits that mounted an unusually strong nAb response against a tier 1 Clade AE virus (TH023.6 strain; ID_50_ >43,740). To our knowledge, this panel of mAbs represents the largest collection of antibodies ever generated from a rabbit against HIV-1 gp120. This report is meant to provide an overview, rather than comprehensive characterization of all of the mAbs generated. It will focus primarily on characterization of the V3 loop mAbs.

## Materials and Methods

### Rabbit immunizations and hybridoma generation

The gp120 protein vaccine used was derived from pcDNA-MCON6gp160 (kindly provided by Dr. Beatrice Hahn [41]). A more extensive description is detailed in a previous publication [42]. The gp120 protein was purified from cell culture supernatant using tandem affinity chromatography (Con A Sepharose and Ni-NTA columns) as previously described [44]. Three female New Zealand white rabbits (2.5–3 kg; Charles River) were immunized subcutaneously with gp120 formulated with Zn-chitosan on weeks 0, 3, 9, 15 and 27 as previously described [42]. One rabbit (gp120-R2) with the highest nAb titer was boosted at week 65. On week 76, the animal was injected intravenously with 1 mg of gp120 in PBS to increase efficiency of generating hybridomas. Four days later, spleen was collected for fusion. All of the studies conducted were approved by IACUC at Iowa State University (#10-09-6772-LM).

The fusion was performed as previously described [40] with a few minor modifications. Briefly, rabbit splenocytes and the fusion partner cell line 240E-1 (kindly provided by Dr. Katherine L. Knight [40]) were fused at a ratio of 2:1 with 50% PEG 1500 (Sigma-aldrich P7181). The hybridomas were selected by growing in media containing HAT (hypoxanthine, aminopterin, and thymidine) (Sigma-aldrich H0262). Hybridoma supernatants were collected and screened for gp120 binding by ELISA, and for neutralization activity as described below. Hybridomas that were positive for gp120 binding were cloned by limiting dilution, expanded and frozen at -140°C for future use.

### ELISA with proteins or overlapping peptides

ELISAs were performed as previously described [42,43] with some modifications. For determination of antibody titer and screening of our hybridoma panel, the indicated proteins were coated onto 96-well Nunc-Immuno plates overnight at 4°C at 30 ng per well using an antigen coating buffer (15 mM Na_2_CO_3_, 35 mM NaHCO_3_, 3 mM NaN_3_, pH 9.6). For screening hybridomas for the inner domain peptides (ID-P) and fine epitope mapping of V3 loop positive antibodies, 15-mer linear overlapping peptides were coated onto 96-well Nunc-Immuno Plates overnight at 4°C at 20 pmol per well in the same antigen coating buffer. Uncoated surfaces were blocked with 200 μl of a blocking buffer (PBS, pH 7.4, containing 2.5% skim milk and 5% calf serum) for 1 h at 37°C. Wells were subsequently washed five times with a wash buffer (PBS containing 0.1% Tween 20) using a Biotek automated plate washer. For antibody titer, all rabbit sera were serially diluted in blocking buffer as indicated in figures, and 100 μl was added to each well. For the cross reactivity of our panel of hybridomas and V3 fine epitope mapping, all the hybridoma supernatants were diluted 1:2 with blocking buffer and 100 μl was added to the each well. For the reactivity of hybridomas to ID-P, 100 μl of the hybridoma supernatant was added directly to each well. The rest of the procedure was similar to what has been described before [42,43]. All assays were done in duplicate.

The 15-mer overlapping peptide set for gp120 based on the M group consensus sequence (CON-S) was obtained from the NIH AIDS Reagent Program (Cat# 9487). V3 and V5 peptides based on the MCON6 sequence (H-TRPNNNTRKSIHIGPGQAFYATGEIIGDIRQAH-OH and H-GNNSNKNKTETFRPG-OH, respectively) were synthesized commercially by CHI Scientific (Maynard, MA). Peptides were coated onto wells at 20 pmol per well.

### Neutralization assays

Virus neutralization assays were done using single cycle HIV-1 pseudovirus infections of TZM-bl cells as described elsewhere [42,45,46]. Briefly, heat inactivated rabbit sera (56°C for 1hr), hybridoma supernatant or purified IgG was diluted in 100 μl of cell culture media (DMEM supplemented with 10% heat inactivated FBS and 1% penicillin/streptomycin). Test samples were diluted over a range of 1:20 to 1:43740 in cell culture medium and pre-incubated with virus (~150,000 relative light unit equivalents) for 1 hr at 37°C before addition of cells. Following a 48 hr-incubation, cells were lysed and Luc activity determined using a microtiter plate luminometer and BriteLite Plus Reagent (Perkin Elmer). Neutralization titers are the sample dilution (for serum) or antibody concentration (for sCD4, purified IgG preparations and monoclonal antibodies) at which relative luminescence units (RLU) were reduced by 50% compared to RLU in virus control wells after subtraction of background RLU in cell control wells.

### Competition assays

Competition assays were performed by modifying the previously described method [43]. The amount of coating antigen used was 30 ng per well. Briefly, equal amount of hybridoma supernatant was added to equal amount of blocking buffer. This initial dilution was then subjected to two-fold serial dilutions. Monoclonal antibodies were diluted to a concentration of 1 μg/ml in blocking buffer. 50 ul of the diluted antibody was added to each well along with 50 μl of the serially diluted hybridoma supernatant. Hence the final monoclonal antibody concentration during the assay was 0.5 μg/ml in each well, and the starting supernatant dilution was 1:4. In order to test competition at a higher concentration, 50 μl of hybridoma supernatant was added directly with 50 μl of competing antibody (at 1 μg/ml concentration) to result in the 1:2 dilution. Antibodies used for competition included VRC01 [8], and PGT121 [47]. For competition of purified monoclonal antibody to PGT121, 10A37 was diluted to a concentration of 6 μg/ml in blocking buffer and then subjected to two-fold serial dilutions. 50 μl of the diluted PGT antibody was added to each well along with 50 μl of the serially diluted 10A37 antibody. Hence, the final PGT concentration was 0.5 μg/ml in each well, and the starting 10A37 concentration was 3 μg/ml. Another mAb (2C2) that was isolated from a rabbit immunized with C-terminal 54 amino acid of gp41 ectodomain (to be published elsewhere), was used at similar concentrations as a negative control. VRC01 or PGT121 was detected using HRP-conjugated, Fc-specific, anti-human antibody as previously described [43].

### Cloning of hybridoma antibody genes

Total RNA was extracted from hybridomas using the RNeasy Mini kit (Qiagen) using the Qiacube automated platform. Following extraction, RNA samples were treated with DNAse (Invitrogen) to remove genomic DNA. Samples were subjected to cDNA synthesis using random hexamers and SuperScript III Reverse Transcriptase (Invitrogen). Briefly, 2 μL of random hexamers (Roche) and 2 μL of 10 mM dNTPs were added to 22 μL of DNAse treated RNA (equivalent to approximately 3x10^6^ cells). The mixture was heated to 65°C for 5 min, then cooled briefly on ice. Subsequently, 8 μL of 5x First-Strand Buffer, 2 uL of 0.1 M DTT, 2 uL of RNaseOUT (Invitrogen), and 2 uL of SuperScript III were added to the mixture. Reaction was incubated at 25°C for 5 min, 45°C for 45 mins, and 70°C for 15 mins. Resulting cDNA was subjected to Antibody gene amplification using Platinum Pfx polymerase (Invitrogen) according to manufacturer’s recommendations. Primers for Antibody gene amplification were based on a previous publication [48]. Primers used for heavy chain amplification were 5’- AGGAATTCTGCAGCTCTGGCACAGGAGCTC-3’ and 5’- CTCCGGATCCGTCGACAGGACTCACCACCTGAGGAGACGGTGACCA-3’. Primers used for kappa chain amplification were 5’- TATCCGTGCACTCCACCATGGACACGAGGGCCCCCACT-3’ and 5’- GTTAGATCTATTCTACTCACGACCTTTGACCACCACCTCGGTCCCTCCGCCGAA-3’ or 5’- TCACTGGCGGTGCCCTGGCAGGCGTCT-3’ (10A37 only). Cycling conditions were as follows: Initial denaturation at 94°C for 5 mins; followed by 35 cycles of 94°C for 30 sec, 68°C for 1.5 mins; final extension at 68°C for 7 mins; hold at 4°C. Resulting PCR products were directly sequenced. Alternatively, the 10A3 and 10A37 hybridomas were subjected to Antibody gene specific cDNA generation and PCR using the SuperScript III One-Step RT-PCR System (Invitrogen), using the primers described.

### Heavy and light chain sequence analysis

Heavy and kappa chain sequences were analyzed with IMGT/V-quest [49] to determine germline usage, mutations present, and CDR domain lengths. Protein sequence alignments were performed with Clustal Omega (www.ebi.ac.uk/Tools/msa/clustalo/).

### Expression and purification of 10A3 and 10A37 antibodies

Antibody variable regions were cloned into either the pFUSEss-CHIg-hG1 and pFUSEss-CLIg-hk (human conserved regions, 10A3 heavy and kappa chain respectively, InvivoGen) or pFUSEss-CHIg-rG and pFUSEss-CLIg-rk2 (rabbit conserved regions, 10A37 heavy and kappa chain respectively, InvivoGen) vectors for expression. Heavy chain primers for 10A3 were 5’- ACGTGAATTCGCAGGAGCAGCTGGAGGAGTC-3’ and 5’- GACCGCTAGCTGAGGAGACGGTGACCAG-3’. Kappa chain primers for 10A3 were 5’- GGTCGAATTCAGCTCAAGTGCTGACCCAG-3’ and 5’-GACCCGTACGTTTGACCACCACCTCG-3’. Heavy chain primers for 10A37 were 5’-ACGTGAATTCGCAGGAGCAGCTGGTGGAGTC-3’ and 5’-GCCCACTCGAGACGGTGACCAGGGTGCCTGGGC-3’. Kappa chain primers for 10A37 were 5’-GGCGAATTCAGCCCTTGTGATGACCCAG-3’ and 5’-CGAGCTAGCTCGCTCTAACAGTCACCCCTATTG-3’. Restriction sites introduced for subsequent cloning are underlined. The heavy chain PCR product for 10A3 and vector were digested with EcoRI and NheI. The kappa chain PCR product for 10A3 and vector were digested with EcoRI and BsiWI. The heavy chain PCR product for 10A37 and vector were digested with EcoRI and XhoI. The kappa chain PCR product for 10A37 and vector were digested with EcoRI and NheI. Standard ligation protocols generated the final 10A3 rabbit-human chimera and 10A37 rabbit expression vectors, and sequencing confirmed an in frame variable region fusion.

For 10A3 and 10A37 antibodies purification, heavy and kappa chain constructs were co-transfected into freestyle 293F cells with 293fectin (Invitrogen). The supernatant was collected 5 days after transfection and clarified by centrifugation, followed by immobilized protein A affinity chromatography purification (Pierce). Purified 10A3 and 10A37 was dialyzed in PBS (pH 7.4), aliquoted and then stored at -80°C.

## Results

### Antibody responses against gp120 booster immunization following an extended resting period

**Fig 1:**
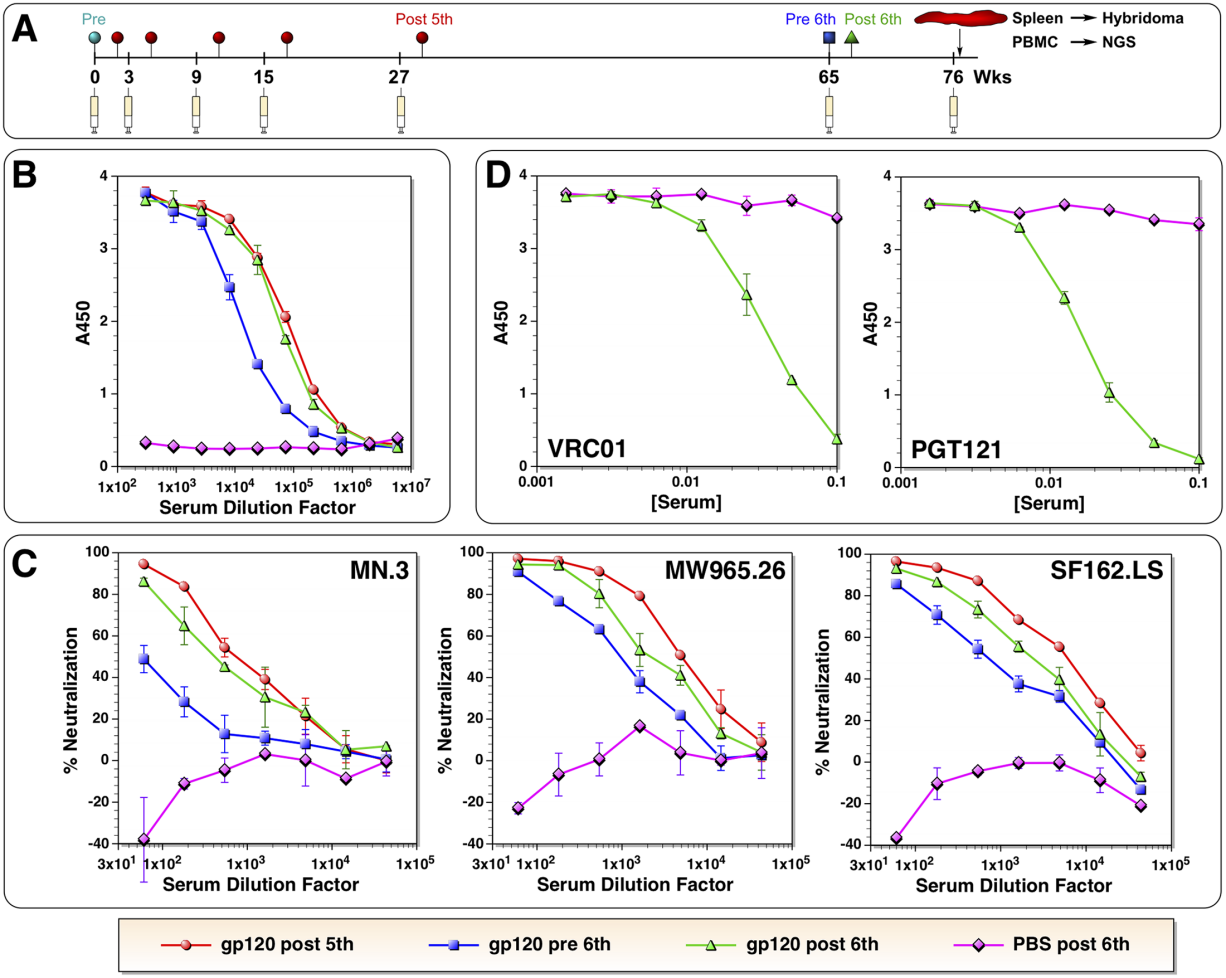
Characterization of antibodies induced after the 6^th^ immunization. (A) Timeline of immunization and sample collection. (B) Comparison of antigen-specific antibody titers after the 5^**th**^, before 6^**th**^, and after 6^**th**^ immunization. A serum sample from an age matched, mock-immunized animal, indicated as “PBS”, was used as a negative control. A450 represents absorbance value at 450 nm. The same legend at the bottom is used for all of the panels. (C) Neutralizing activity against MN.3, MW965.26 and SF162.LS. (D) The serum sample after the 6th immunization was tested for competition against VRC01 and PGT121. The values on the x-axis represent serum concentration (1/dilution factor).

In previous reports, we described antibody responses against monomeric MCON6 gp120 in rabbits following five immunizations over a period of about 29 weeks [42,43]. We selected one of the animals (rabbit #2) that had mounted strong neutralizing activity against Clade AE, tier 1 TH023.6 isolate (ID_50_ >43,740 in TZM-bl assay), as well as some activity against tier 2 isolates, for long-term evaluation. The animal was allowed to rest for 38 weeks and immunized a 6^th^ time on week 65 (Fig 1A). A serum sample was collected just prior to immunization (referred to as “pre 6^th^”) to assess durability of antibody responses and to determine the baseline level, and two weeks post immunization on week 67 (referred to as “post 6^th^”) to evaluate recall responses.

Antibody levels were monitored by ELISA using the autologous antigen (Fig 1B). A serum sample collected two weeks after the fifth immunization (on week 29) was used for direct comparison. A serum from an age-matched, mock-immunized rabbit (designated as “PBS”) was used as a negative control. Results showed that the antibody level declined approximately 7–8 fold during the 38 weeks of resting period after the fifth immunization (estimated half-life of about 12.7 weeks). However, after the sixth immunization, the antibody level increased back to the level achieved after the fifth immunization, which appeared to be the maximum achievable antibody titer. Immunogenic linear epitope profile, as determined by ELISA using 15-mer overlapping peptides, was largely similar to that observed after the fifth immunization (S1 Fig; [43]).

As described previously [42,43], the potency of neutralizing activity induced in this rabbit was quite substantial, albeit largely against tier 1 viruses. More interestingly, antibodies that could compete with bnAbs VRC01 as well as PGT121 and PGT126 in binding gp120 were observed [43]. Although we did not detect broadly neutralizing activity against tier 2 viruses, we hypothesized that better understanding of gp120-induced antibodies could allow for better design of future immunogens as well as immunization strategies. Our goal was to characterize these antibodies at a monoclonal level. Because such characterizations are time consuming and laborious, we first examined whether the rabbit maintained nAbs as well as VRC01- and PGT121-competing antibodies before undertaking a detailed molecular analysis.

Neutralizing activity of sera from pre- and post-6^th^ immunization was tested against tier 1A viruses from Clade B (MN.3 and SF162.LS) and Clade C (MW965.26). Serum sample after the 5^th^ immunization was also tested for comparison. As expected, neutralizing activity declined against all three viruses during the resting period between the fifth and the sixth immunization (Fig 1C), similar to the decline observed in antibody titers (Fig 1A). Upon the sixth immunization, serum neutralization increased, but remained at a slightly lower level than what was observed after the fifth immunization. It should be noted that significant enhancement of pseudovirus infectivity was observed in mock-immunized serum when used at high concentrations (less than 1:100 dilution; shows up as negative neutralization). The reason for this phenomenon is not yet known. Antibody competition analyses also indicated that antibodies that could compete with bnAbs VRC01 and PGT121 in binding gp120 were also maintained (Fig 1D), albeit at a slightly lower level compared to the level observed after the fifth immunization [43]. All together, these results indicated that despite slight reductions in neutralizing activity and antibody levels that compete with bnAbs, the overall quality of antibody responses remained largely unchanged despite a prolonged resting period. Based on these results, we decided to further characterize antibody responses by generating mAbs.

### Generation of hybridomas and epitope mapping analyses by ELISA

To generate hybridomas, the rabbit was injected intravenously with 1 mg of soluble gp120 in PBS without adjuvants at week 76. Four days later, splenocytes were harvested for generating hybridomas. From ten 96-well plates used for the fusion, 548 hybridomas were generated. Hybridoma supernatants were collected and screened for gp120 binding by ELISA. In total, 95 clones were gp120-specific (17.3% cloning efficiency) although two were lost during propagation.

**Fig 2:**
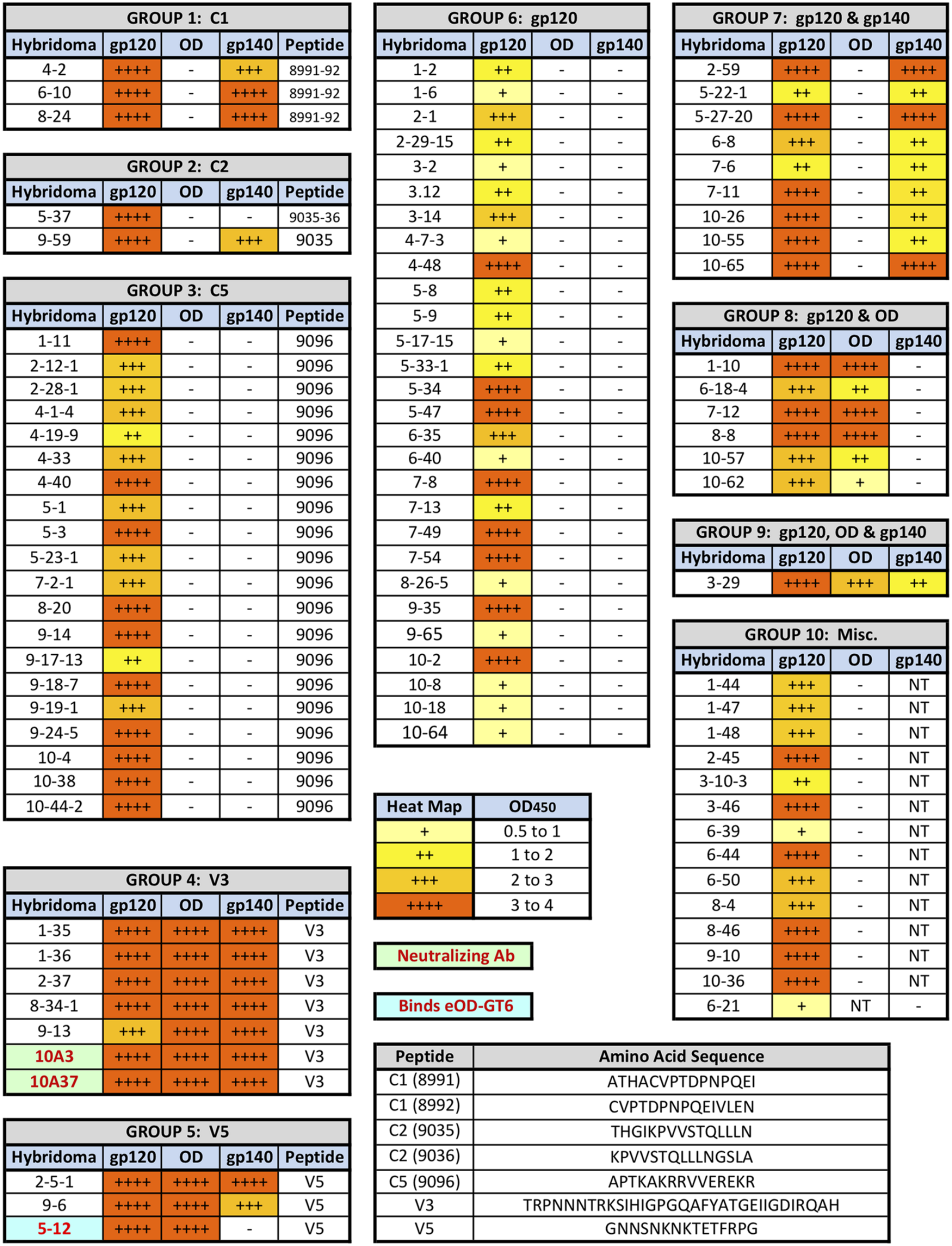
Epitope mapping analyses of hybridomas generated. Hybridomas were evaluated for reactivity against gp120, gp120-OD, BG505 SOSIP gp140, as well as eOD-GT6 and a large panel of peptides. Hybridomas are arranged in groups based on their linear epitopes or their reactivity to three proteins. NT: Not Tested.

With a long-term goal of establishing a vaccine-induced “antibodyome” database for gp120, we initiated an effort to define the epitopes recognized by the mAbs by doing ELISA with hybridoma culture supernatant. Besides gp120, reactivity against three other envelope-derived protein constructs were evaluated, including gp120-OD (MCON6 gp120 outer domain we reported recently; [42]), BG505 SOSIP gp140 (soluble, stable trimeric envelope; [50,51]), and eOD-GT6 (engineered outer domain that can bind germline BCR of VRC01; [52]). To narrow down epitopes, many peptides that were shown to be highly immunogenic were used (S1 Fig; [43]). Results from these ELISA are summarized in Fig 2. Linear epitopes for 35 of 93 mAbs were identified: Group 1 (C1), group 2 (C2), group 3 (C5), group 4 (V3) and group 5 (V5). The rest of the mAbs, epitopes of which could not be defined to short linear peptides, were grouped based on their reactivity against gp120, gp120-OD and gp140.

**Fig 3:**
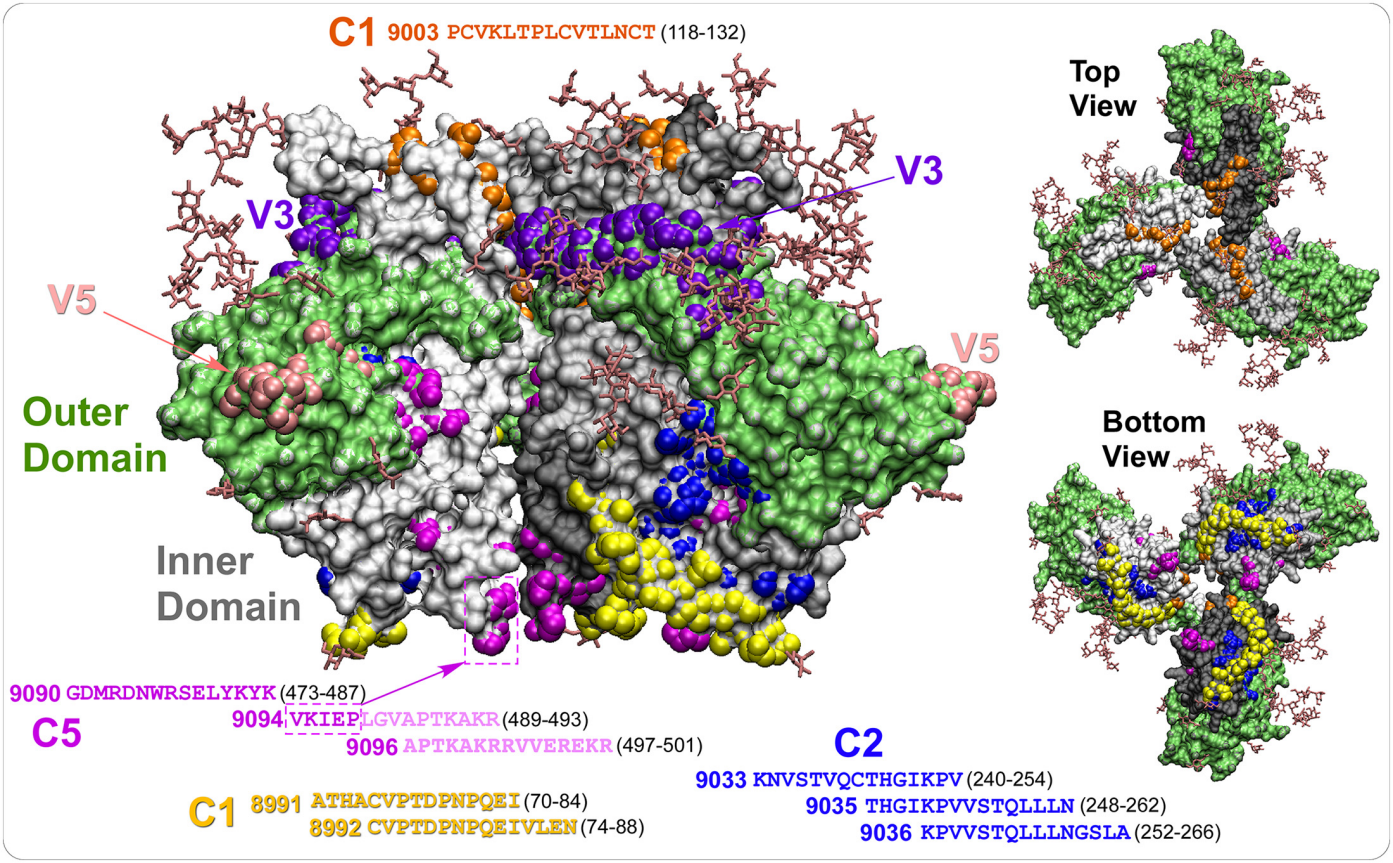
Locations of immunogenic peptides in the inner domain. A crystal structure of trimeric BG505 SOSIP gp140 (pdb: 4NCO) was used to illustrate locations of the immunogenic peptides. Only the gp120 portion is shown for clarity. The outer domain is shown in lime and the inner domain is shown in three shades of gray. A part of peptide 9094 and 9096 from the C5 region (indicated in a lighter magenta shade) is not shown in the crystal structure. The arrow points to the position of the five amino acids (VKIEP) on peptide 9094. For simplicity, the locations of the V3 and V5 loops are shown only on the side view.

Out of 35 mAbs with known linear epitopes, 20 were against peptide 9096, which is located at the very C-terminal end of gp120 in the C5 region, indicating that it is extremely immunogenic on soluble gp120. Five other mAbs were directed against epitopes in the inner domain: Three against C1 (peptides 8991 and 8992) and two against C2 (peptides 9035 and 9036). Interestingly mAb 5–37 reacted to both peptides 9035 and 9036 while mAb 9–59 bound only peptide 9035. In addition, only 9–59 bound gp140, indicating a clear difference between the two mAbs in the amino acid residues being recognized and/or the angle of approach in epitope binding. Although peptide 9003, located just upstream of the V1 loop, was also immunogenic, we did not isolate any mAbs directed against the peptide. In the outer domain of gp120, seven mAbs (1–35, 1–36, 2–37, 8-34-1, 9–13, 10A3 and 10A37) were directed against the V3 loop and three mAbs (2-5-1, 9–6 and 5–12) bound the V5 loop peptide. To facilitate visualization, a number of immunogenic epitopes are plotted onto the crystal structure of SOSIP gp140 (Fig 3).

**Fig 4:**
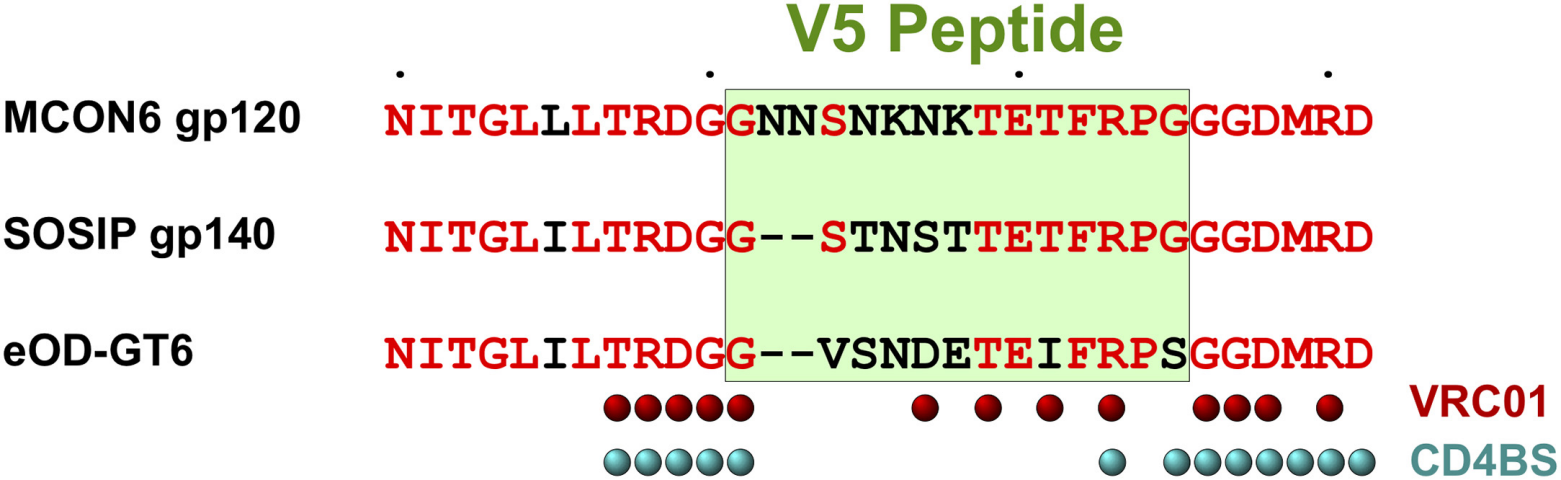
Sequence alignment of a region around the V5 loop. Sequences for the three antigens used for ELISA are shown (MCON6 gp120, BG505 SOSIP gp140 and eOD-GT6). The V5 loop peptide used for ELISA is boxed in. Identical amino acid residues are indicated in red. The residues that make contact with bnAb VRC01 or CD4 are indicated as red or cyan circles.

There were seven mAbs that bound gp120 and gp120-OD, but not SOSIP gp140 (mAbs in group 8 and mAb 5–12 in group 5). Overall, MCON6 and BG505 share 81% amino acid sequence identity. Thus, one possibility is that amino acid sequences between MCON6 and BG505 are different at these epitopes. Alternatively, the epitopes might not be accessible on the trimeric SOSIP gp140. Interestingly, 5–12 was the only mAb that could bind eOD-GT6. Moreover, it was the only V5 mAb that failed to bind gp140. Surprisingly, amino acid sequence alignment analyses of the region around the V5 loop showed that SOSIP gp140 sequence is actually more homologous to MCON6 gp120 than is eOD-GT6 (Fig 4). At the present time, it is not known why 5–12 mAb binds to MCON6 gp120, gp120-OD and eOD-GT6, but not SOSIP gp140.

### Neutralizing activity and competitive binding to or near the neutralizing epitopes

**Fig 5:**
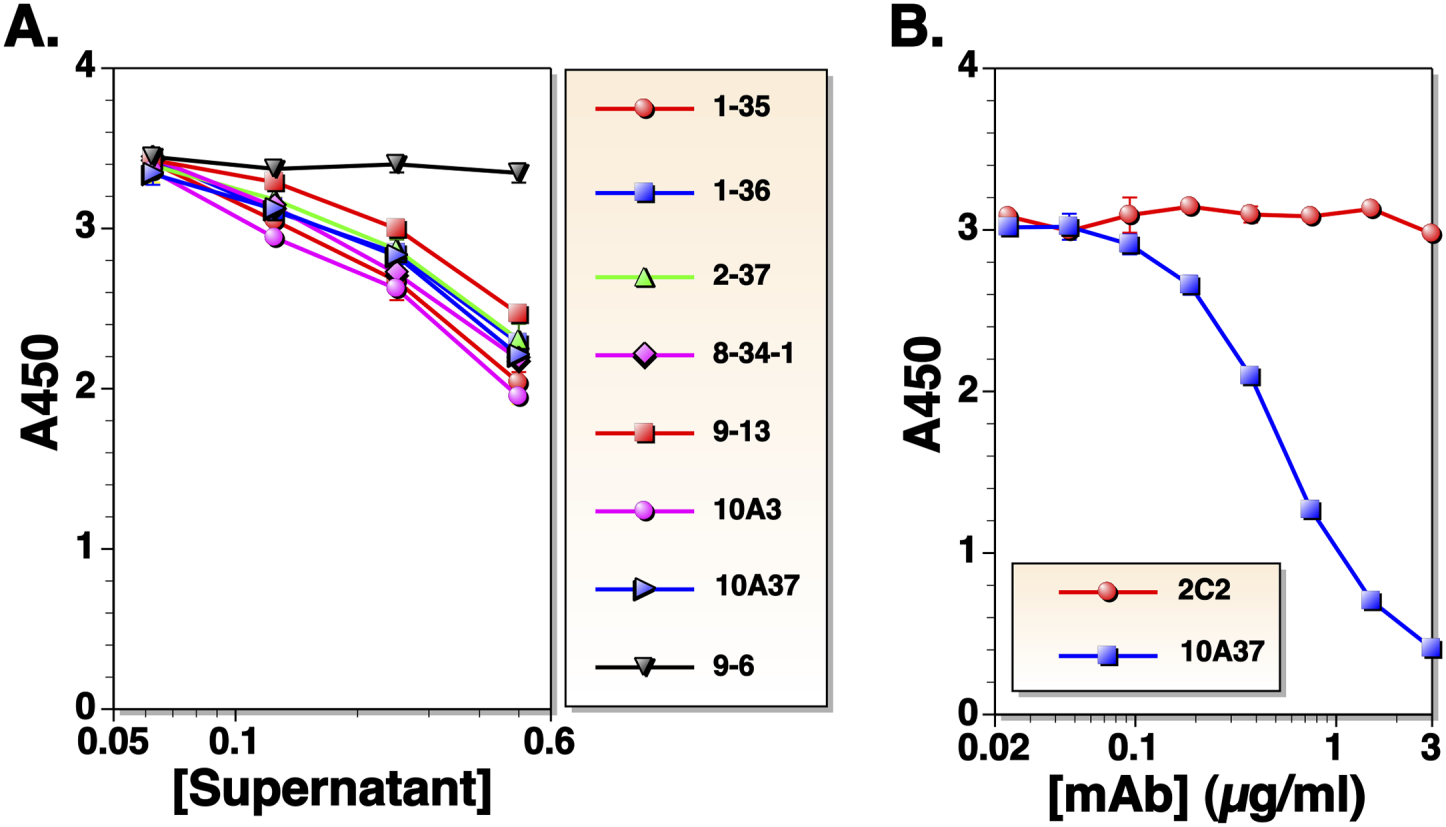
Antibody competition against PGT121. (A) Culture supernatants of hybridomas specific against the V3 loop were evaluated for competing activity against PGT121 for binding gp120. The values on the x-axis represent supernatant concentration (1/dilution factor). (B) Recombinant mAb 10A37 was used for the competition assay. Anti-gp41, recombinant mAb 2C2 was used as a negative control.

Since the serum from the rabbit showed significant competition against VRC01 and PGT121 (Fig 1D), we screened all hybridomas for competing activities against these bnAbs. Unfortunately, we did not detect any hybridomas that could compete against VRC01 for gp120 binding, suggesting that such antibodies might be rare. Given that only a small fraction of splenocytes likely have yielded hybridomas, it is probable that B cells expressing VRC01 competing antibodies were not incorporated into our panel. While screening for mAbs that could compete against PGT121, we found seven hybridomas that possessed low, but definite activity (Fig 5A). Interestingly, all of the competing hybridomas were reactive against the V3 loop peptide (Fig 2) suggesting that serum competition observed against PGT121 might be due to the strong anti-V3 antibody response. Finally, we screened all the hybridomas for neutralizing activity against tier 1 pseudoviruses SF162.LC and MW965.26. Culture supernatants from two V3 peptide positive hybridomas, 10A3 and 10A37, showed strong neutralizing activity (see below).

### Detailed characterization of anti-V3 loop mAbs

**Fig 6:**
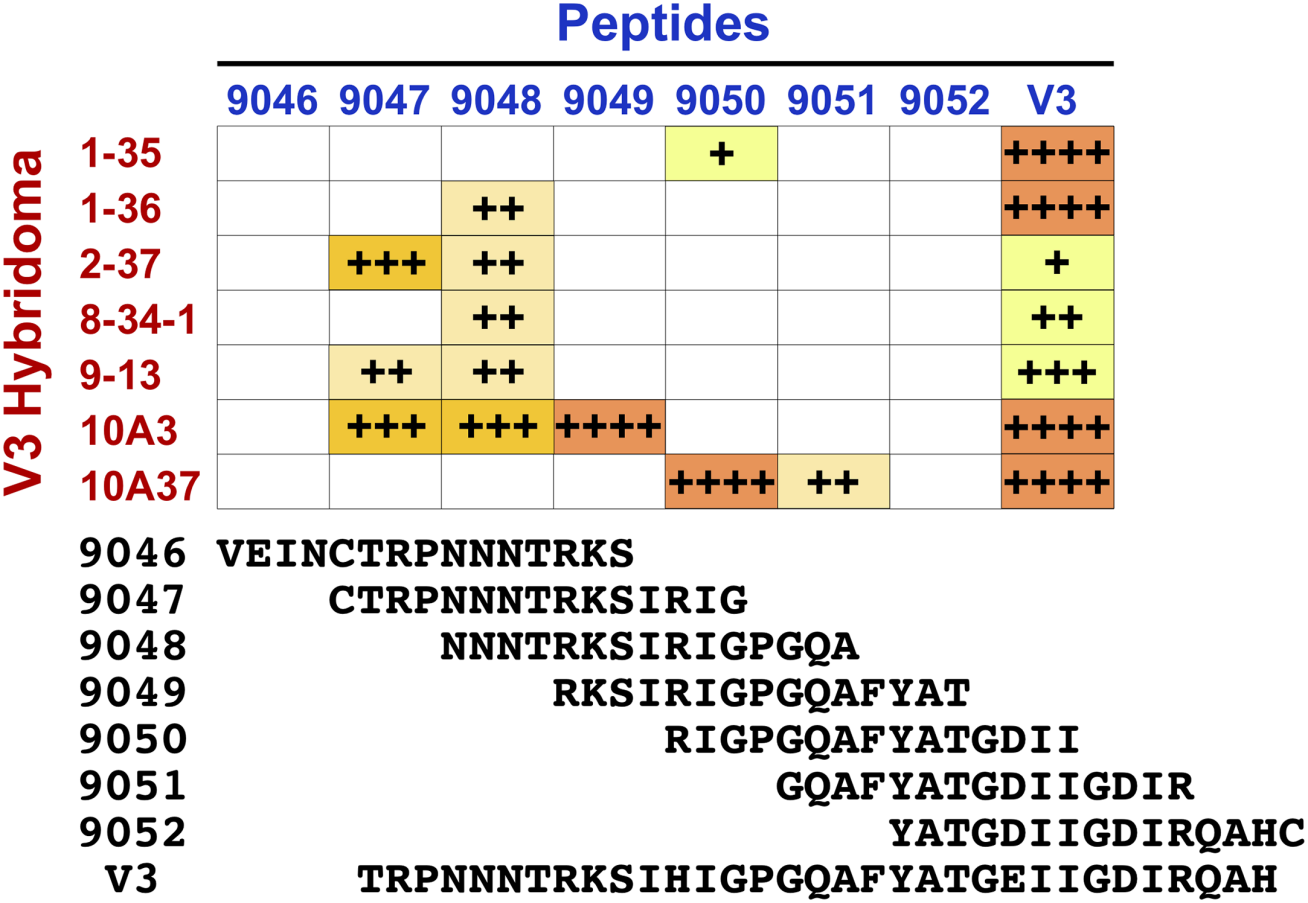
Epitope mapping analyses of anti-V3 loop mAbs. The V3 loop-positive antibodies were tested for binding to overlapping 15-mer peptides spanning the entire loop by ELISA. The sequences of the peptides are shown at the bottom.

Since all anti-V3 loop mAbs could compete against PGT121 and two of them exhibited neutralizing activity, all of them were further characterized. First, fine epitope mapping analysis was done using overlapping 15-mer linear peptides that span the entire length of the V3 loop (Fig 6). The ELISA result demonstrated that peptide 9048 (NNNTRKSIRI**GPGQ**A) was the most immunoreactive segment in the V3 loop, as five mAbs could interact with this peptide. This is consistent with an observation that N-terminal half of the V3 loop is more immunogenic than the C-terminal half [42,43]. mAbs 1–36 and 8-34-1 recognized only peptide 9048. Another pair, 2–37 and 9–13, bound to peptides 9047 (CTRPNNNTRKSIRI**G**) and 9048. Hybridoma 1–35 was only weakly positive to peptide 9050 (RI**GPGQ**AFYATGDII). The two hybridomas that were identified as having neutralizing activity, 10A3 and 10A37, exhibited totally different peptide recognition patterns. 10A3 reacted most strongly against peptide 9049 (RKSIRI**GPGQ**AFYAT), but also recognized peptides 9047 and 9048. The peptide 9049 has the tip of the V3 crown (**GPGQ**) almost exactly at the center. This would suggest that mAb 10A3 has the profile of many other anti-V3 loop neutralizing mAbs, such as 447-52D [53], and HGN194 [54]. In contrast to 10A3, 10A37 strongly reacted against peptide 9050 and moderately to peptide 9051 (**GQ**AFYATGDIIGDIR). Out of the seven mAbs, 10A37 was the only one that recognized peptide 9051, indicating that recognition of C-terminal half of the V3 loop is indeed rare. In this regard, 10A37 is a novel V3 loop mAb.

**Fig 7:**
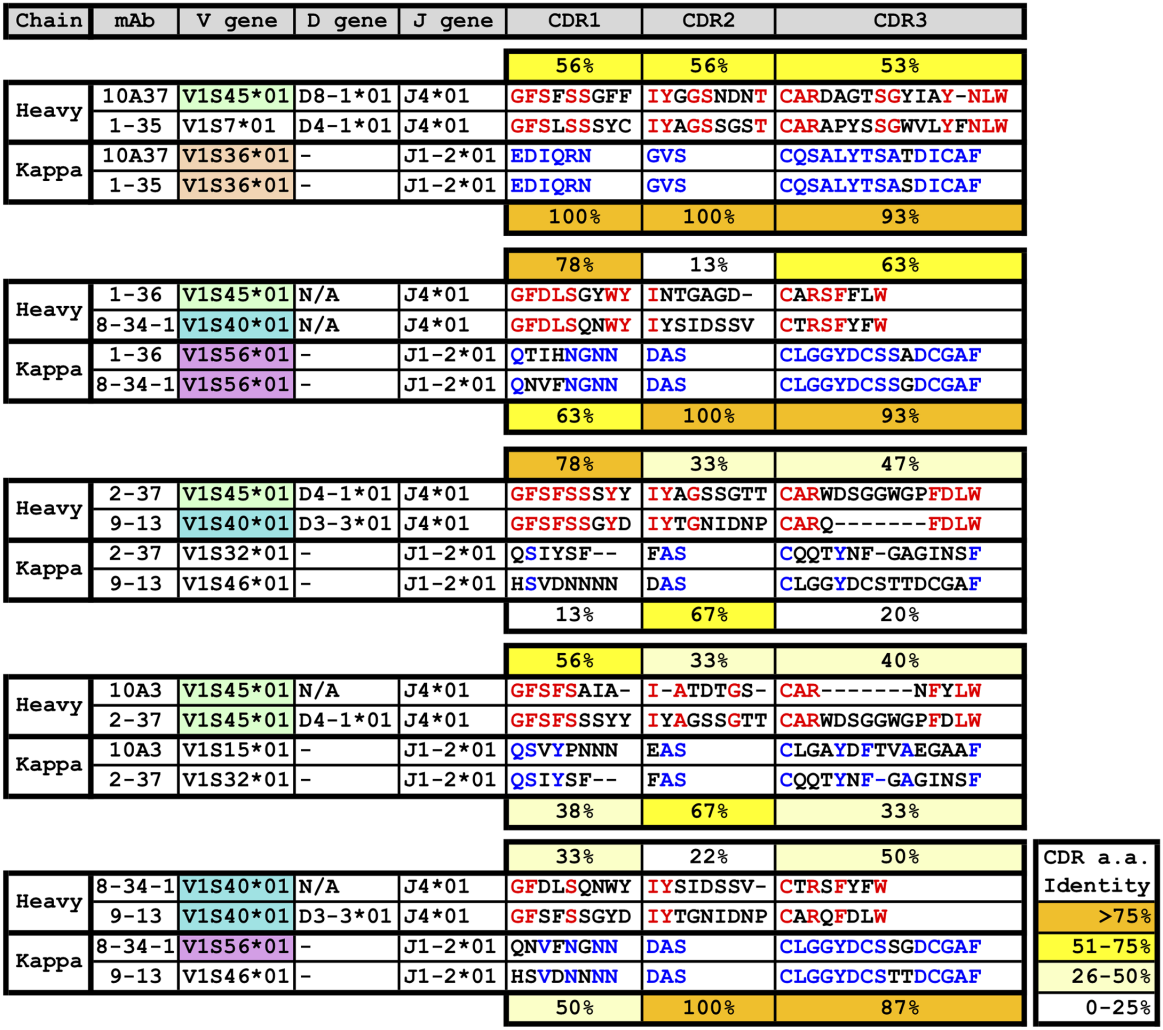
Comparison of CDR regions of the anti-V3 loop mAbs. The heavy and light chains of the seven V3 loop-positive mAbs were aligned for analysis. Comparison was done based on peptide reactivity shown in Fig 6. Percentages indicate % amino acid identity between the two CDR being compared.

To further characterize V3 mAbs at the molecular level, antibody genes were RT-PCR amplified from hybridomas, sequenced and analyzed using IMGT/V-QUEST database [49]. First, many of the mAbs utilized the same germline V genes (Fig 7, S2 Fig). Four of the mAbs (10A37, 1–36, 2–37 and 10A3), including the two that exhibited neutralizing activity used V1S45*01 V_H_ gene. mAbs 8-34-1 and 9–13 were derived from V1S40*01. There were two pairs of mAbs that used the same Vk gene (V1S36*01 for 10A37 and 1–35, and V1S56*01 for 1–36 and 8-34-1). Interestingly, the light chain of mAbs 10A37 and 1–35, both of which bound peptide 9050, were nearly identical. In contrast, HCDR sequences, which were derived from different germlines, were only about 53–56% identical.

Similar to 10A37 and 1–35, mAbs 1–36 and 8-34-1, both of which only bound peptide 9048, were derived from the same V1S56*01 V_k_ gene. Amino acid identities for LCDR1, -2 and -3 were 63%, 100% and 93%, respectively. Even though their V_H_ genes were derived from different germlines, their HCDR1 and HCDR3 sequences were quite similar (78% and 63%, respectively). Conversely, mAbs 10A3 and 2–37, both of which bound peptides 9047 and 9048, originated from the same V1S45*01 V_H_ germline, but their HCDR1, -2 and -3 sequences were markedly different (only 56%, 33% and 20% identity, respectively). These differences might have enabled 10A3 to bind peptide 9049 strongly and exhibit neutralizing activity.

### Characterization of recombinant neutralizing mAbs

**Fig 8:**
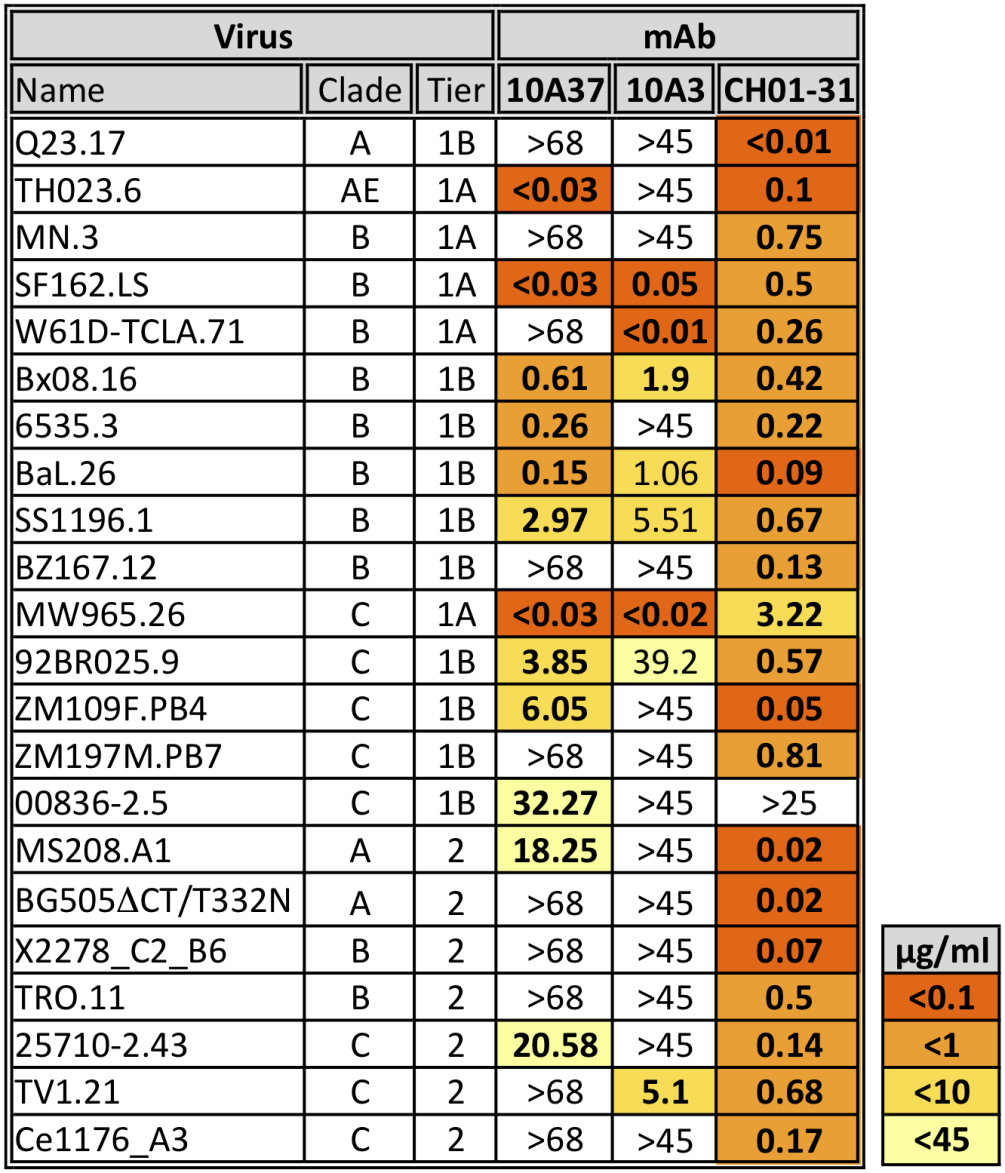
Neutralizing activity of anti-V3 loop mAbs 10A3 and 10A37. V3 positive mAbs 10A37 and 10A3 were tested for neutralization against pseudoviruses belonging to different clades and tiers of HIV-1 and their IC_50_ values (μg/ml) are shown. These mAbs were compared to a combination of two mAbs (CH01 and VRC-CH31) that are known to show broad cross-clade neutralization [56].

To further characterize the neutralizing activity, recombinant mAbs 10A3 and 10A37 were cloned, expressed and purified. Their potency and breadth were assessed using a standard TZM-bl neutralization assay against a large panel of tier 1 and 2 pseudovirus from different clades (Fig 8). Recombinant mAb 10A3 neutralized several Clade B and Clade C tier 1A and 1B isolates, as well as one tier 2 virus Clade C virus (TV1.21). Neutralizing potency and breadth of mAb 10A37 was more impressive than 10A3, being able to neutralize several additional viruses. In particular, 10A37 exhibited potent neutralizing activity against Clade AE virus TH023.6, which was shown to be highly susceptible to the immune sera shown in previous report [42]. In addition, 10A37 was able to neutralize tier 2 Clade C virus 25710–2.43, which is one of the twelve virus isolates that belong to the a new “global panel of reference strains” [55]. Unfortunately, 10A37 did not neutralize any of the other eleven viruses from the “global panel” (*i*.*e*. IC_50_ >25 μg/ml). 10A37 and 10A3, combined, were able to neutralize 11 of 15 tier 1A and 1B viruses tested (73%) and only 3/18 tier 2 viruses (17%). However, they were insufficient to recapitulate full neutralizing activity of antibodies present in the immune serum, which was able to neutralize MN.3 [42].

In Fig 5A, we showed that all of the anti-V3 loop mAbs exhibited ability to compete with PGT121 for binding gp120. To confirm that anti-V3 loop antibodies are indeed able to compete with PGT121, we used recombinant 10A37. As shown in Fig 5B, 10A37 was able to efficiently compete with PGT121 for binding gp120. These results suggest that PGT121-competing activity we observed in immune sera could actually be due to anti-V3 antibodies, rather than true PGT121-like antibodies that bind V3 and a glycan immediately adjacent to the C-terminus of V3.

## Discussion

The field of HIV-1 vaccine development has been aided immensely by the recent discovery of new bnAbs [11]. Furthermore, deep sequencing analyses have highlighted the complex evolution that the immunoglobulin germline must undergo to generate these rare antibodies [57,58]. Additional antibody structural studies, especially when performed in complex with HIV-1 envelope glycoproteins, have provided valuable insights for generating rationally designed immunogens [50–52]. However, translating the information generated from these studies into designing immunogens that can elicit similar bnAbs has been difficult. A critical fact that needs to be kept in mind is that HIV-1 infected individuals provide an environment where the virus and the immune system responses co-evolve. This dynamic environment generated by chronic virus infection is difficult to replicate using any vaccination regimen. Hence, while characterization of antibody responses in virus-infected individuals is valuable, immunization studies in animal models remain a vital means to evaluate realistic vaccine strategies against HIV-1.

Of all HIV-1 Env immunization studies conducted in animal models, only a limited number of studies have attempted to further characterize antibody responses at a clonal level. Generating mAbs is a labor intensive and time-consuming process. However, they can significantly aid in understanding the immune response as demonstrated in this study. First, mAbs permit precise mapping of B cell epitopes, which would not be possible using polyclonal antisera. This would provide necessary information to establish a comprehensive map of vaccine-induced antibodyome against HIV-1 envelope glycoprotein, which we believe will be critical for developing a vaccine that can induce bnAbs against the virus. In this study, as an initial attempt to define epitopes recognized by what we believe is the largest panel of anti-gp120 rabbit mAbs generated to date, we examined antibody reactivity against different protein constructs and some of the immunogenic peptides identified from overlapping peptide ELISA [43]. Characterization of epitope targets would greatly aid our understanding of immune responses against HIV-1. This report focused primarily on V3 loop-specific mAbs since two of them exhibited potent neutralizing activity against tier 1 viruses with marked breadth.

The V3 loop has been known as the principal neutralizing determinant for over two decades [59,60]. Despite the fact that nAbs targeting this epitope exhibit only a limited breadth and largely against tier 1 viruses, they are the only ones that could be induced consistently in both animals and humans in a vaccine setting. It should be noted, however, that not all anti-V3 loop neutralizing mAbs exhibit equal potency or breadth; while some only neutralize autologous vaccine strains, others do exhibit marked breadth. Most of the broadly neutralizing V3 loop mAbs characterized to date have been derived from virus-infected human patients, including 447-52D that was reported over 20 years ago [53]. Recently, Hioe *et al*. [60] tested the breadth and potency of seven human anti-V3 loop antibodies. Their study showed that 56/98 (57%) psuedoviruses tested (both tier 1 and 2 isolates from Clades A, AG, B, C and D) could be neutralized by one or more mAbs (using Area Under Curve-methodology). More importantly, 9/24 (37.5%) tier 2 viruses could be neutralized by one or more mAbs. In another study, Corti *et al*. [54] characterized an anti-V3 mAb HGN194 in direct comparison to 447-52D. While 447-52D could neutralize only 88% of the tier 1 and 4% of the tier 2 viruses tested, HGN194 was able to neutralize all tier 1 and 11% of the tier 2 viruses, suggesting superior breadth.

In contrast to mAbs generated from virus-infected humans, there are far fewer mAbs generated from animals immunized with HIV-1 antigens, especially those that exhibit neutralizing activity. Recently, however, Chen *et al*. reported the isolation of twelve mAbs from a rabbit immunized with a DNA prime-protein boost JR-FL gp120 vaccine regimen [39]. One of the antibodies recovered, R56, targeted the V3 loop and neutralized multiple tier 1 viruses belonging to Clades B, C, AE and AG as well as two tier 2 viruses in standard TZM-bl assays. It is not easy to compare neutralization potency or breadth of different mAbs characterized in different laboratories because not all of the same viruses are tested and the assays are not performed with identical virus stocks. Having said that, the potency of 10A37 seemed to be greater than those of R56. For example, 10A37 neutralized five viruses that R56 could not, including MS208.A1 (Clade A), 6535.3 (Clade B), ZM109F.PB4, 00836–2.5 and 25710–2.43 (Clade C). The neutralization potency (IC_50_) of 10A37 was also significantly greater than R56 for multiple viruses: TH023.6 (<0.03 vs. 18.57 μg/ml)), SF162.LS (<0.03 vs. 0.1), Bal.26 (0.15 vs. 2.07), Bx08.16 (0.61 vs. 3.18), SS1196.1 (2.97 vs. 7.18). The only virus R56 neutralized, but could not by 10A37 was TV1.21 (Clade C). However, 10A3 could neutralize TV1.21 with greater potency than R56 (5.1 vs. 25.63 μg/ml). Surprisingly, none of the three rabbit mAbs could neutralize MN.3, which is generally considered to be a highly sensitive tier 1 isolate. While there is a known structural basis for the failure of R56 to neutralize MN.3 [61], the reason for the lack of neutralization by 10A3 and 10A37 remains to be determined. Interestingly, neither 10A3 nor 10A37 neutralized BG505ΔCT/T332N, despite the fact that these two mAbs strongly reacted to SOSIP gp140 by ELISA (Fig 2).

One of the advantages of mAbs is that they allow functional separation of antibodies with different properties. As such, one of our objectives was to isolate mAbs that could compete against bnAbs VRC01 or PGT121 in binding gp120. Unfortunately, we were unable to identify any VRC01-competing mAbs. On the other hand, we identified seven mAbs that could compete with PGT121 and these turned out to be specific for the V3 loop. This result indicates that PGT121-competing activity we detected in immune sera [43] is likely due to a high level of antibodies against the V3 loop. This finding highlights a possibility that induction of high titers of V3 loop antibodies might prevent eliciting more effective PGT121-like antibodies. As such, it might be prudent to minimize immunogenicity of the V3 loop in future immunogen design.

One other important benefit of working with mAbs is the ability to evaluate antibody repertoire and function at the molecular level. This is particularly useful when evaluating multiple mAbs that target nearby epitopes at a given region (*e*.*g*. V3 loop), yet exhibit different functional phenotypes (*e*.*g*. neutralizing vs. non-neutralizing). Neutralizing mAb 10A37 and non-neutralizing mAb 1–35 are good examples. These mAbs target the same epitope (*i*.*e*. RIGPGQAFYATGDII), albeit with different affinity. Although their light chains originated from the same germline and are virtually identical in sequence, their heavy chain sequences are quite divergent, which would indicate that the differences in their heavy chains likely account for the phenotypic difference. It should be emphasized that mAbs we generated likely represent only a small subset of all antibodies that could bind any given epitope. As such, they provide only a snapshot of a long evolutionary pathway. Sequence profiling of all the expressed antibodies in the immunized animal using NGS would provide the means by which to examine differences in the maturation pathways of neutralizing versus non-neutralizing antibodies without the limitations imposed by hybridoma production.

Assuming that all B cells have an equal chance of being fused into hybridomas and that specificity of antibodies have no affect on survivability of the hybridomas, the frequency of hybridomas targeting a given epitope should represent the relative immunogenicity of the epitope. In this regard, the single most frequent epitope recognized by the mAbs is the 9096 peptide (20 of 93 mAbs), which we had previously identified as the single most immunogenic peptide based on ELISA with antisera. The 9096 peptide lies at the very C-terminus of gp120. The next most immunogenic epitope is the V3 loop. Considering that antibodies that bind these epitopes are either non-neutralizing or neutralizing with limited breadth, a better immunogen or a vaccine strategy would be needed to improve focusing immune responses towards epitopes targeted by bnAbs. Although we have not succeeded in inducing bnAbs in this study using MCON6 gp120, the methodologies we have established and the reagents we have generated should facilitate evaluation of antibody responses against other immunogens or vaccine strategies in the future.

## Conclusions

In this study, antibody responses against MCON6 gp120 in a rabbit were further characterized at a clonal level using a large panel of monoclonal antibodies (mAbs) generated from the immunized animal. Epitopes were defined using a set of different envelope protein constructs as well as linear peptides. The most immunogenic epitope was at the C-terminal end of the protein, followed by the V3 loop. Two new neutralizing antibodies (nAbs) against the V3 loop were isolated (10A3 and 10A37). While 10A3 was similar to many previously isolated neutralizing mAbs recognizing the N-terminal half of the V3 loop including the crown at the tip, 10A37 was atypical with its epitope positioned more towards the C-terminal half of the loop. 10A37 exhibited potent neutralizing activity with substantial breadth against multiple clades of HIV-1 that exhibit a tier 1A and tier 1B neutralization phenotype. To our knowledge, it is the most potent and broadly neutralizing anti-V3 loop mAb isolated from a vaccinated animal or human to date. Further characterization of its structure as well as the epitope it binds could provide important insights into the neutralization mechanism.

## Supporting Information

S1 Fig Identification of immunogenic linear epitopes by ELISA using overlapping peptides. ELISA was conducted with antisera collected from Rabbit #2 after the sixth immunization using overlapping peptides. Peptide numbers represent catalog numbers from the NIH AIDS Reagent Program. A schematic diagram of gp120 is shown on top. A450 represents absorbance value at 450 nm.
(TIFF)

S2 Fig Amino acid sequence alignments of the anti-V3 mAbs. Alignments are shown for the V-regions of the (A) heavy and (B) kappa chains of the seven antibodies. Framing regions (FR) and complementary determining regions (CDR, red text) are shown, with amino acid conservation indicated below the alignment: “*” identical, “: “ highly similar, “. “ slightly similar. Alignments were performed in Clustal Omega (http://www.ebi.ac.uk/Tools/msa/clustalo/).
(TIFF)
