# Supplementary figures and images for: Characterization of a Large Panel of Rabbit Monoclonal Antibodies against HIV-1 gp120 and Isolation of Novel Neutralizing Antibodies against the V3 Loop
Source: PLoS One. 2015 Jun 3;10(6):e0128823. doi: 10.1371/journal.pone.0128823 (PMC4454676; doi:10.1371/journal.pone.0128823)

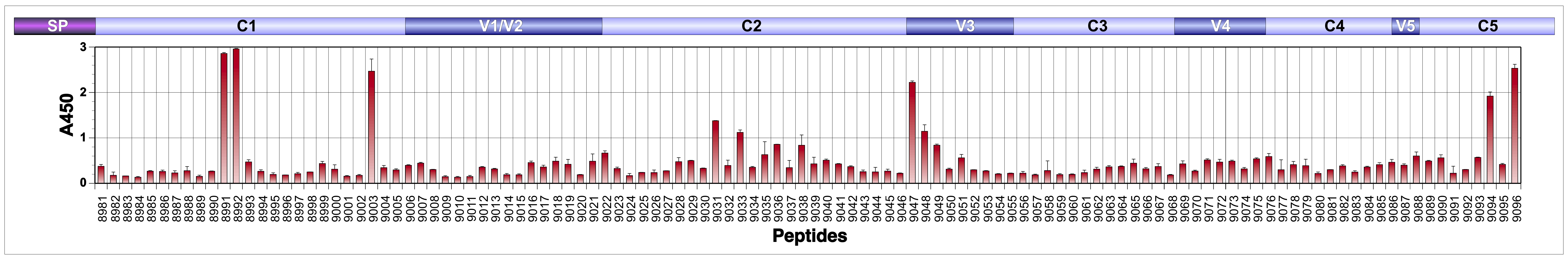

Supplement: S1 Fig — ELISA was conducted with antisera collected from Rabbit #2 after the sixth immunization using overlapping peptides. Peptide numbers represent catalog numbers from the NIH AIDS Reagent Program. A schematic diagram of gp120 is shown on top. A450 represents absorbance value at 450 nm. (TIFF) [file pone.0128823.s001.tiff]

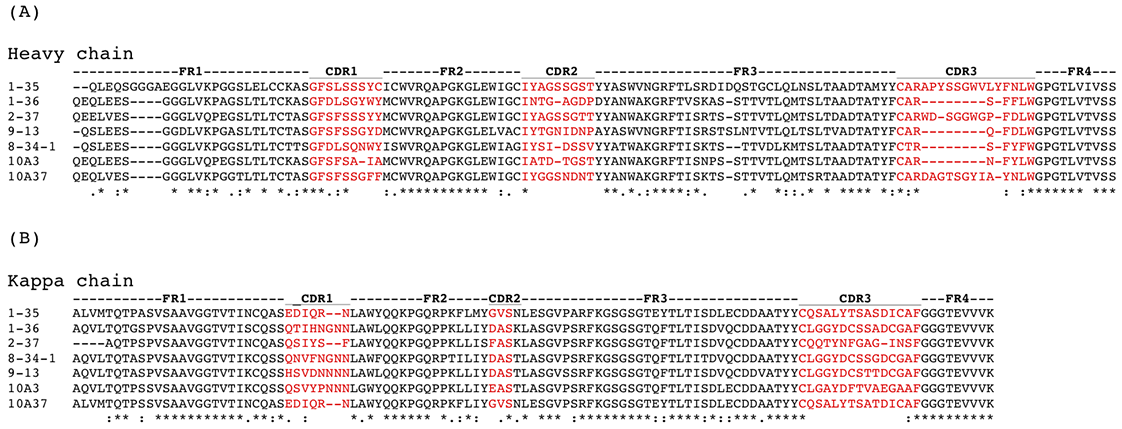

Supplement: S2 Fig — Alignments are shown for the V-regions of the (A) heavy and (B) kappa chains of the seven antibodies. Framing regions (FR) and complementary determining regions (CDR, red text) are shown, with amino acid conservation indicated below the alignment: “*” identical, “: “ highly similar, “. “ slightly similar. Alignments were performed in Clustal Omega (http://www.ebi.ac.uk/Tools/msa/clustalo/). (TIFF) [file pone.0128823.s002.tiff]
